# Relationship between *Helicobacter pylori* and glycated hemoglobin: a cohort study

**DOI:** 10.3389/fcimb.2023.1196338

**Published:** 2023-06-09

**Authors:** Yi Chen, Chaoyu Yang, Ningning You, Jinshun Zhang

**Affiliations:** ^1^ Departments of Gastroenterology, Taizhou Hospital of Zhejiang Province Affiliated to Wenzhou Medical University, Linhai, Zhejiang, China; ^2^ Health Management Center, Taizhou Hospital of Zhejiang Province Affiliated to Wenzhou Medical University, Linhai, Zhejiang, China

**Keywords:** *Helicobacter pylori*, triglyceride glucose index, insulin resistance, cohort study, glycated hemoglobin

## Abstract

**Background:**

*Helicobacter pylori* (*H. pylori*) has increasingly been shown to be related to extragastric diseases. Glycated hemoglobin A1c (HbA1c), an indicator of glycemic control, is closely linked to the event of diabetes. The purpose of this research was to analyze the association between *H. pylori* and HbA1c through a cohort study.

**Methods:**

The population who underwent multiple physical checkups in the physical examination center of Taizhou Hospital was included. All of them underwent urea breath test, serological examination and physical parameter measurement. Multiple regression was used for analyzing the influencing factors of HbA1c. In addition, the result of HbA1c on *H. pylori* infection was studied by restricted cubic spline (RCS) analysis. The triglyceride glucose (TyG) index represents the level of insulin resistance (IR) in the population. The population was classified on the basis of primary and last *H. pylori* infection, therefore, the variations of HbA1c and TyG index among totally different teams were investigated.

**Results:**

Multiple regression demonstrated that *H. pylori* was an influential factor in HbA1c. RCS analysis showed a nonlinear relationship between HbA1c and *H. pylori* infection. When HbA1c>5.7%, the chance of *H. pylori* infection was considerably enlarged. Additionally, long-term *H. pylori* infection increased HbA1c levels, while HbA1c levels decreased after *H. pylori* eradication. Similarly, long-term *H. pylori* infection also increased the TyG index.

**Conclusion:**

Prediabetes increases the danger of *H. pylori* infection, long-term *H. pylori* infection increases HbA1c and IR levels, and wipeout of *H. pylori* could have a positive impact for glycemic control in the population.

## Introduction

*Helicobacter pylori* (*H. pylori*), a chronic infection of the gastrointestinal tract, has a higher prevalence in developing countries and is a global public health problem (Zamani et al., 2018). *H. pylori* can make functional dyspepsia, chronic gastritis, gastric ulcers, and even gastric cancer (McColl, 2010). In recent years, there are more and more studies on *H. pylori* and extra-gastric diseases, such as dermatological, cardiovascular, neurological, immune, respiratory, blood system, and metabolic diseases (Wong et al., 2014; Yu et al., 2019; Santos et al., 2020; Wang et al., 2020; Doheim et al., 2021; Reuter et al., 2023). *H. pylori* can turn out a variety of inflammatory factors, like C-reactive protein, interleukin, tumor necrosis factor α (TNF- α), and reduce the level of leptin (Noach et al., 1994; Perri et al., 1999; Chen et al., 2021). High levels of inflammatory factors and leptin deficiency are key mechanisms of insulin resistance (IR) and metabolic syndrome (Longo-Mbenza et al., 2007; Li et al., 2022).

Diabetes is a chronic metabolic disease with a rising global prevalence and a huge economic burden (Yazdanpanah et al., 2017). Glycated hemoglobin A1c (HbA1c) ≥6.5% has been included in the diagnostic criteria for diabetes mellitus, representing last 2 or 3 months of glycemic control (American Diabetes Association, 2009; Hinzmann et al., 2012). IR plays a crucial part in a variety of diseases, including diabetes and cardiovascular disease (Stumvoll et al., 2005; Wang et al., 2018; Adeva-Andany et al., 2019). Hyperinsulinemic-Euglycemic Clamp (HIEC) is that the gold criterion for designation of IR, however, owing to its complexity and high cost, it is difficult to be introduced in the physical examination population (Cersosimo et al., 2014). The triglyceride glucose (TyG) index is a novel clinical alternative index for IR (Guerrero-Romero et al., 2010; Lee et al., 2016). Because of its simplicity, accessibility and reproducibility, it has received much attention in risk and diagnostic studies for the development of type 2 diabetes (Lee et al., 2014; Wang et al., 2021).

However, the connection between *H. pylori* and HbA1c as well as IR is controversial. In a cross-sectional study involving 37,263 people, *H. pylori* infection was associated only with dyslipidemia, not fasting blood glucose (FBG) or HbA1c (Kim et al., 2016). In addition, *H. pylori* infection failed to take issue between diabetic and non-diabetic populations, and *H. pylori* eradication had no result on blood glucose and IR (Park et al., 2005; Demir et al., 2008). However, in some researches, *H. pylori* infection has been related to glycemic management in diabetic populations, and wipeout of *H. pylori* may benefit glycemic management (Cheng et al., 2019; Song et al., 2021).

Given the unspecified association between *H. pylori* and HbA1c, the research explored the influence of *H. pylori* infection on HbA1c and IR through a large cohort study.

## Materials and methods

### Data collection

In this study, people who underwent physical examination at the Health Examination Center of Taizhou Hospital between 2017 and 2022 were included. The age, sex, smoking and alcohol history, laboratory indicators and carbon breath test results of the patients were collected. Laboratory parameters included triglyceride (TG), total cholesterol (TC), high-density lipoprotein (HDL), low-density lipoprotein (LDL), FBG and HbA1c. We excluded patients who lacked complete clinical information or who had a history of malignancy, severe cardiovascular disease, or gastrointestinal surgery. All people had undergone multiple medical examinations, with an interval of >6 months between the first and last examination. A total of 9,266 individuals participated in the study.

### Clinical indicators collection

Trained nurses inquired the patient’s age, gender, smoking and drinking history, and measured diastolic blood pressure (DBP) and systolic blood pressure (SBP) in the quiet state. After fasting for 8 hours during the night, venous blood was collected from people under fasting condition for testing laboratory parameters such as TC, TG, LDL, HDL, FBG, and HbA1c. Glycated hemoglobin analyzer was used to measure HbA1c levels in the population. The calculation formula of TyG index was [Tg (mg/dL) ×FBG (mg/dL)/2] (Guerrero-Romero et al., 2010).

### Detection of *Helicobacter pylori*

*H. pylori* was detected by ^13^C or ^14^C urease breath tests (Fischbach and Malfertheiner, 2018). The process of the ^13^C breath test was as follow: the first breath sample was collected in the fasting state, then the capsule labeled with ^13^C was taken, the respiratory sample was collected after waiting 30 minutes, and finally the two samples were analyzed on the apparatus. The procedure of the ^14^C breath test was as follow: taken a ^14^C urea capsule and added water, waited for 15 minutes, evenly blown air through the conduit for 1-3 minutes, and obtained the results by inserting the gas collecting card into the detector.

### Statistical analysis

For continuous variables, Mann-Whitney test or independent T test were used to analyze the differences between groups. Categorical variables were tested by chi-square test and expressed as counts and percentages. Multiple regression was used to analyze the influencing factors of HbA1c. Additionally, we investigated the link between HbA1c and *H. pylori* using restricted cubic spline (RCS) analysis with nodes set at the 5th, 35th, 65th, and 95th percentiles. Statistical analysis was performed in R software (version 4.1.3), and p<0.05 was deemed significant.

## Result

### Baseline characteristics

The baseline characteristics of the population were shown in **Table 1**. All medical examiners were divided into *H. pylori* negative and positive groups depending on breath test. The positive group was more likely to be male, older, and included more smokers than negative group. Additionally, compared to negative group, positive group had higher HbA1c and lower HDL levels (p<0.05).

**Table 1:** Baseline characteristics of all physical examination populations.

Variables	*H. pylori* negative (n=5328)	*H. pylori* positive (n=3938)	p-value
Gender (n, %)			0.020
Female	1925 (36.1)	1331 (33.8)	
Male	3403 (63.9)	2607 (66.2)	
Smoke (n, %)			0.003
No	3997 (75.0)	2847 (72.3)	
Yes	1331 (25.0)	1091 (27.7)	
Drink (n, %)			0.136
No	4300 (80.7)	3129 (79.5)	
Yes	1028 (19.3)	809 (20.5)	
Age (year)	48.33 ± 11.78	49.33 ± 11.82	<0.001
Triglycerides (mmol/L)	1.87 ± 1.59	1.92 ± 1.67	0.133
Total cholesterol (mmol/L)	5.04 ± 0.96	5.03 ± 0.95	0.950
High density lipoprotein (mmol/L)	1.42 ± 0.30	1.40 ± 0.29	0.009
Low density lipoprotein (mmol/L)	2.69 ± 0.73	2.68 ± 0.71	0.264
Diastolic blood pressure (mmHg)	76.07 ± 11.74	77.38 ± 11.71	0.215
Systolic blood pressure (mmHg)	126.53 ± 17.46	127.18 ± 17.98	0.080
Fasting blood glucose (mmol/L)	5.38 ± 1.43	5.42 ± 1.48	0.128
Glycated hemoglobin A1c (%)	5.83 ± 0.87	5.89 ± 0.90	0.004

### Multiple regression analysis

In order to study the factors affecting the level of HbA1c, multiple regression was conducted for gender, age, *H. pylori* infection, smoking, drinking, lipids, blood pressure, and glucose. In the results, *H. pylori* remained the hazard factor for HbA1c (p=0.025), **Table 2**.

**Table 2:** Multiple regression analysis.

Variables	β	p-value
Gender (%)	-0.028	<0.001
Smoke (%)	0.043	<0.001
Drink (%)	0.010	0.095
Age (>50)	0.099	<0.001
*H. pylori* infection (+)	0.012	0.025
Triglycerides (mmol/L)	-0.020	0.060
Total cholesterol (mmol/L)	0.060	0.006
High density lipoprotein (mmol/L)	-0.061	<0.001
Low density lipoprotein (mmol/L)	-0.011	0.568
Diastolic blood pressure (mmHg)	-0.042	<0.001
Systolic blood pressure (mmHg)	0.008	0.337
Fasting blood glucose (mmol/L)	0.834	<0.001

### Nonlinear relationship between HbA1c and *H. pylori* infection

**Figure 1:**
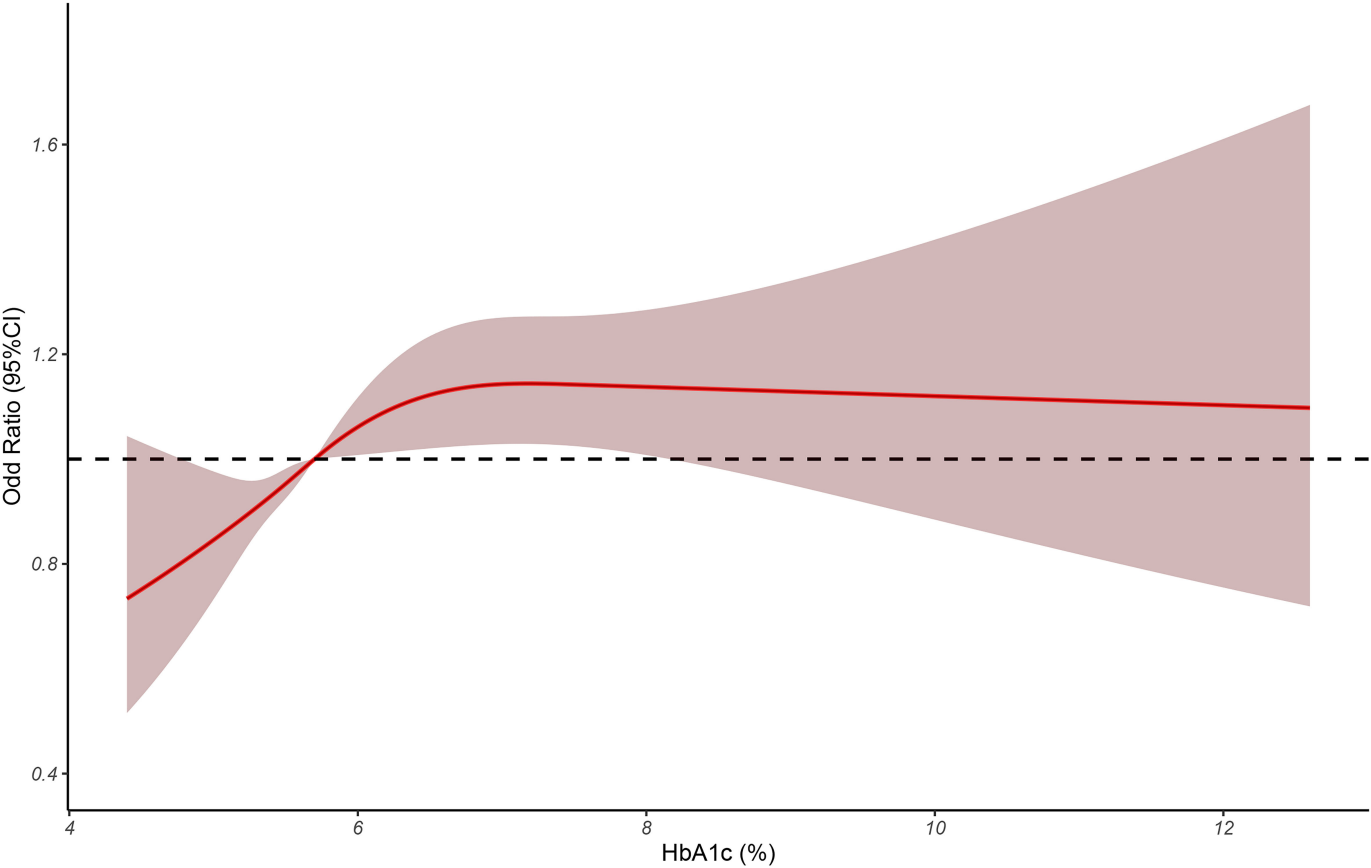
The restricted cubic spline was used to analyze the relationship between HbA1c and *H. pylori* infection.

We used RCS model to evaluate the relevance between HbA1c and *H. pylori* infection. As shown in **Figure 1**, when HbA1c>5.7%, the hazard of *H. pylori* infection elevated markedly with the increase of HbA1c (p<0.05).

### Longitudinal association between HbA1c and *H. pylori* infection

**Figure 2:**
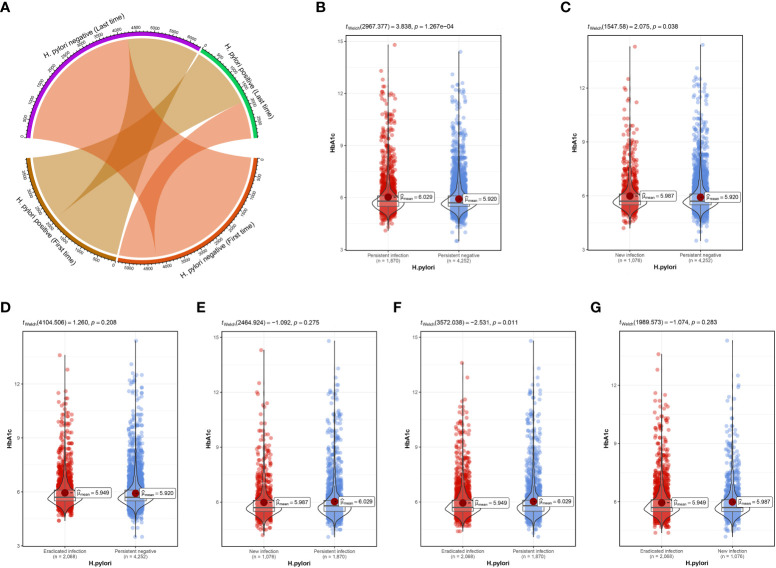
**(A)** Changes in the status of first and last *H pylori* infections. **(B)** Difference in HbA1c between persistent infection and persistent negative. **(C)** Difference in HbA1c between new infection and persistent negative. **(D)** Difference in HbA1c between eradicated infection and persistent negative. **(E)** Difference in HbA1c between new infection and persistent infection. **(F)** Difference in HbA1c between eradicated infection and persistent infection. **(G)** Difference in HbA1c between eradicated infection and new infection.

The median follow-up time for the study population was 1.77 years. Based on status of *H. pylori* infection at primary and last physical examination, all people were divided into *H. pylori* persistent infection, persistent negative, new infection and eradicated infection groups. During the follow-up period, the first and last *H. pylori* infection status changes were shown in **Figure 2A**. Compared with persistent negative group, HbA1c in persistent infection and new infection groups was remarkably higher, p<0.05 (**Figure 2B, C**). The difference between the eradicated infection and the persistent negative groups was not statistically significant, p=0.208 (**Figure 2D**). Further, there was no vital distinction in HbA1c between the persistent infection and the new infection groups (**Figure 2E**), but HbA1c in the eradicated infection group decreased significantly (**Figure 2F**). In addition, no marked difference in HbA1c was seen between new infection and eradicated infection groups, **Figure 2G**.

### Relationship between HbA1c and TyG index

**Figure 3:**
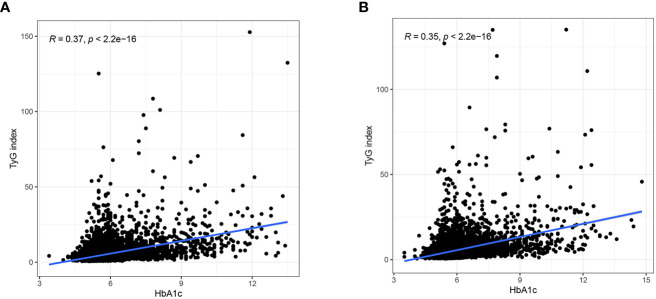
**(A)** Relationship between HbA1c and TyG index at the first physical examination. **(B)** Relationship between HbA1c and TyG index at the last physical examination.

TyG index can represent insulin resistance, and we further studied the link between HbA1c and IR. At the first physical examination, there was a correlation between HbA1c and TyG index (r=0.37), **Figure 3A**. Similarly, at the last physical examination, HbA1c was still significantly correlated with TyG index (r=0.35), **Figure 3B**.

### Effect of *H. pylori* infection on TyG index

**Figure 4:**
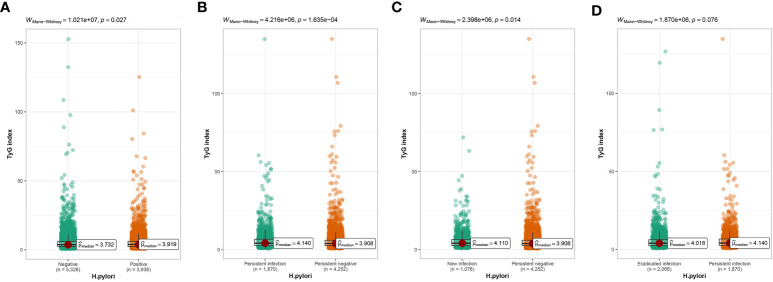
**(A)** Difference in TyG index between *H pylori* negative and positive groups. **(B)** Difference in TyG index between persistent infection and persistent negative. **(C)** Difference in TyG index between new infection and persistent negative. **(D)** Difference in TyG index between eradicated infection and persistent infection.

According to cross-sectional research, we observed that TyG index was considerably higher within *H. pylori* positive group than that in negative group (**Figure 4A**). In longitudinal study, the TyG index in the persistent infection group was apparently increased compared to the persistent negative group (**Figure 4B**). In the same way, TyG index in the new infection group was obviously increased than the persistent negative group (**Figure 4C**). The TyG index in eradicated group showed a downward trend compared with that in persistent infection group, but no difference on statistics (**Figure 4D**).

## Discussion

Diabetes is a chronic metabolic disease whose progression can result in a series of complications such as retinopathy, cardiovascular-related problem, and neuropathy (Cole and Florez, 2020). Blood glucose control is essential in the management of diabetes to delay the emergence and progression of its complications (Vilsbøll et al., 2018; Wang and Hng, 2021). HbA1c, as a serological indicator of glycemic control, is important for preclinical diagnosis of diabetes and prevention of vascular complications (Heianza et al., 2012; Krhač and Lovrenčić, 2019; Halalau et al., 2023). HbA1c between 5.7-6.4% has been used by American Diabetes Association (ADA) as one of the diagnostic criteria for prediabetes (American Diabetes Association, 2021). Poor blood glucose control can lead to an increased prevalence of prediabetes, further increasing the risk of diabetes and its complications (Tabák et al., 2012).

*H. pylori* and diabetes are linked in many aspects, but studies on *H. pylori* and HbA1c are still relatively few, and there are different conclusions. In some meta-analyses, *H. pylori* was not detected to be related to glycemic control in diabetic population (Horikawa et al., 2014). However, in certain studies, *H. pylori* infection was positively correlated to HbA1c levels (Chen and Blaser, 2012). In our study, differences in HbA1c were identified in *H. pylori* negative and positive populations. Nonlinear relationship showed a striking increase in risk of *H. pylori* infection when the HbA1c >5.7%. This means that the population is more susceptible to *H. pylori* infection when there is a tendency towards early diabetes. Our further cohort study confirmed that long-term *H. pylori* infection resulted in apparently elevated HbA1c levels, but after eradication of *H. pylori*, HbA1c levels in the population decreased. It was consistent with some previous studies (Devrajani et al., 2010; Maluf et al., 2020). In addition, in our paper, the IR of the population was significantly higher with the increase of HbA1c. After prolonged *H. pylori* infection, IR levels were significantly higher than that of uninfected population, which may be closely related to the influence of *H. pylori* on glycemic control. However, the mechanisms by which *H. pylori* infection affects glycemic control and IR are complex. Type 1 diabetes mellitus (T1DM) is due to certain factors mediating autoimmune destruction of the pancreas, resulting in insufficient insulin secretion, while type 2 diabetes mellitus (T2DM) is mainly caused by impaired islet function and IR (Ilonen et al., 2019; Bonora et al., 2022). *H. pylori* may modulate the autoimmune response and IR syndrome through chronic inflammation outside the stomach (Polyzos et al., 2011; Marietti et al., 2013; Blosse et al., 2018). It can lead to the destruction of pancreatic islet cells and aggravate insulin secretion deficiency (Wang et al., 2022). In addition, its induced gastritis may affect the abnormal secretion of various hormones and further aggravate IR (Chen et al., 2021). Similarly, abnormal secretion of hormones such as ghrelin and leptin may cause changes in dietary intake, further affecting glycemic control (Aimasso et al., 2019).

Our cohort study, confirmed the nonlinear relationship between *H. pylori* and HbA1c, indicating that *H. pylori* can increase HbA1c levels and have an effect on IR. However, this research still has some limitations. First, it was a single-center study, which may require a multicenter longitudinal study to provide additional evidence. Second, mechanism by which *H. pylori* affects glycemic control is uncertain and perhaps needs to be further explored by biological experiments. Third, there was a lack of analysis of certain confounding factors, such as exercise and obesity, which may affect HbA1c levels (de Moura et al., 2023). In addition, although we did not use the homoeostasis model assessment of IR (HOMA-IR) to measure IR in the physically examined population because fasting insulin levels were not measured, the TyG index has been extensively studied as an alternative indicator of IR (Minh et al., 2021; Lopez-Jaramillo et al., 2023).

## Conclusion

*H. pylori* is closely related to glycemic control in the population, and prediabetic populations may be more likely to be infected with *H. pylori*. Long-term *H. pylori* infection can increase HbA1c levels, worsen glycemic control, and exacerbate IR. Eradication of *H. pylori* has a positive impact on glycemic control in the population.

## Data availability statement

The raw data supporting the conclusions of this article will be made available by the authors, without undue reservation.

## Ethics statement

The studies involving human participants were reviewed and approved by Ethics Committee of Taizhou Hospital (K20220790). Written informed consent for participation was not required for this study in accordance with the national legislation and the institutional requirements.

## Author contributions

JZ made great contributions to the concept, design and data acquisition of the article. YC and CY was mainly involved in data analysis. NY was involved in article writing. All authors contributed to the article and approved the submitted version.
